# Using an onboard kilovoltage imager to measure setup deviation in intensity‐modulated radiation therapy for head‐and‐neck patients

**DOI:** 10.1120/jacmp.v8i4.2439

**Published:** 2007-09-24

**Authors:** James G. Mechalakos, Margie A. Hunt, Nancy Y. Lee, Linda X. Hong, C. Clifton Ling, Howard I. Amols

**Affiliations:** ^1^ Department of Medical Physics Memorial Sloan–Kettering Cancer Center New York New York U.S.A.; ^2^ Department of Radiation Oncology Memorial Sloan–Kettering Cancer Center New York New York U.S.A.

**Keywords:** head and neck, IMRT, IGRT, setup error

## Abstract

The purpose of the present study was to use a kilovoltage imaging device to measure interfractional and intrafractional setup deviations in patients with head‐and‐neck or brain cancers receiving intensity‐modulated radiotherapy (IMRT) treatment.

Before and after IMRT treatment, approximately 3 times weekly, 7 patients were imaged using the Varian On‐Board Imager (OBI: Varian Medical Systems, Palo Alto, CA), a kilovoltage imaging device permanently mounted on the gantry of a Varian 21EX LINAC (Varian Medical Systems). Because of commissioning of the remote couch correction of the OBI during the study, online setup corrections were performed on 2 patients. For the other 5 patients, weekly corrections were made based on a sliding average of the measured data. From these data, we determined the interfractional setup deviation (defined as the shift from the original setup position suggested by the daily image), the residual error associated with the weekly correction protocol, and the intrafractional setup deviation, defined as the difference between the post‐treatment and pretreatment images. We also used our own image registration software to determine interfractional and intrafractional rotational deviations from the images based on the template‐matching method. In addition, we evaluated the influence of inter‐observer variation on our results, and whether the use of various registration techniques introduced differences. Finally, translational data were compared with rotational data to search for correlations.

Translational setup errors from all data were 0.0±0.2 cm, −0.1±0.3 cm, and −0.2±0.3 cm in the right–left (RL), anterior–posterior (AP), and superior–inferior (SI) directions respectively. Residual error for the 5 patients with a weekly correction protocol was −0.1±0.2 cm (RL), 0.0±0.3 cm (AP), and 0.0±0.2 cm (SI). Intrafractional translation errors were small, amounting to 0.0±0.1 cm, −0.1±0.2 cm, and 0.0±0.1 cm in the RL, AP, and SI directions respectively. In the sagittal and coronal views respectively, interfractional rotational errors were −1.1±1.7 degrees and −0.5±0.9 degrees, and intrafractional rotational errors were 0.3±0.6 degrees and 0.2±0.5 degrees. No significant correlation was seen between translational and rotational data.

The OBI image data were used to study setup error in the head‐and‐neck patients. Nonzero systematic errors were seen in the interfractional translational and rotational data, but not in the intrafractional data, indicating that the mask is better at maintaining head position than at reproducing it.

PACS numbers:87.53.Kn, 87.53.Oq

## I. INTRODUCTION

Accurate and repeatable setup of patients is a requisite in radiotherapy. In the treatment of head‐and‐neck tumors, accurate setup is particularly important because of the proximity of the treatment volume to critical structures such as the spinal cord, brainstem, and parotid glands. When intensity‐modulated radiotherapy (IMRT) is used, proximity of this kind between the target and normal organs often leads to highly inhomogeneous fluence profiles with steep dose gradients. Thus, repeatable and accurate patient setup is crucial to prevent the potential hazard of encompassing critical structures—for example, the spinal cord—in the high‐dose region. Indeed, studies have shown that improper setup in the head‐and‐neck region can have significant effects on tumor coverage and normal‐tissue sparing.^(^ ^1^ ^–^ ^3^ ^)^ Improvement of head‐and‐neck setup precision and reproducibility therefore continues to be an active field of study.

One approach to evaluating setup errors is to classify them into two categories: interfractional and intrafractional. The former reflect setup differences from day to day, and the latter, changes in patient position during a treatment session. Setup verification studies more often measure the former^(^ ^4^ ^–^ ^11^ ^)^, but some measure the latter.^(^ ^7^ ^,^ ^10^ ^)^ Measurement of interfractional and intrafractional setup error through the course of treatment therefore requires repeated imaging of the patient on the treatment table. This imaging may result in excessive dose to the patient if megavoltage (MV) imaging is used. Kilovoltage (kV) imaging results in a lower patient dose, and it can therefore be used with sufficient frequency to acquire more setup information per patient than traditional MV imaging allows.

The advent of onboard kV imaging devices has also introduced new and more effective means for measuring and correcting setup error at the treatment machines. These devices not only allow measurement of setup error at the treatment console, but also remote correction of the setup based on the measured error. The advantages of kV over MV imaging for setup correction have been shown to include not only lower dose, but also smaller inter‐observer variability and significantly better setup error reduction in the head‐and‐neck region.^(5)^ Because these imagers are of the projection variety, little information about soft tissue is available, and registration occurs by alignment of bony anatomy. But the imagers can also be used to generate cone‐beam computed tomography (CT) scans if soft‐tissue registration is required.

Our institution recently purchased and commissioned a Varian On‐Board Imager (OBI: Varian Medical Systems, Palo Alto, CA) system for use with a Varian 21EX linear accelerator (Varian Medical Systems). Using this device, we conducted a study of interfractional and intrafractional setup error for 6 patients undergoing IMRT treatment for head‐and‐neck cancer and 1 patient undergoing hypofractionated IMRT treatment to the brain.

For part or all of their treatment course, patients were imaged an average of 3 times weekly, both before and after treatment. Approximately 150 orthogonal image pairs were acquired for this study. Because the OBI unit was new to the department for the bulk of this study, standard weekly megavoltage images were also acquired for these patients as a “gold standard” and were compared to the kV images from the same day for quality assurance purposes. The remote couch correction functionality of the OBI was being tested at the time of the study, and therefore for 5 of the 7 patients, the suggested OBI shift at the machine was not applied but was instead averaged offline to determine a weekly setup shift. For the remaining 2 patients, online correction was carried out.

Here, we report on the setup errors determined from the OBI images for all 7 patients and on the residual error associated with the weekly correction protocol.

## II. METHODS

### A. Translational setup error

The Varian OBI consists of a 125‐kVp X‐ray tube isocentrically mounted to the gantry of the accelerator; it is operated from the treatment console. Orthogonal image pairs acquired by the unit are registered to reference images, and a translational setup correction is suggested. This correction can then be applied remotely by the user. The purpose of the present study was not to illustrate the clinical use of the OBI unit, which was still in the acceptance phase during most of this study. Rather, it was to present the results of a setup error study based on the images acquired by the device.

**Figure 1:**
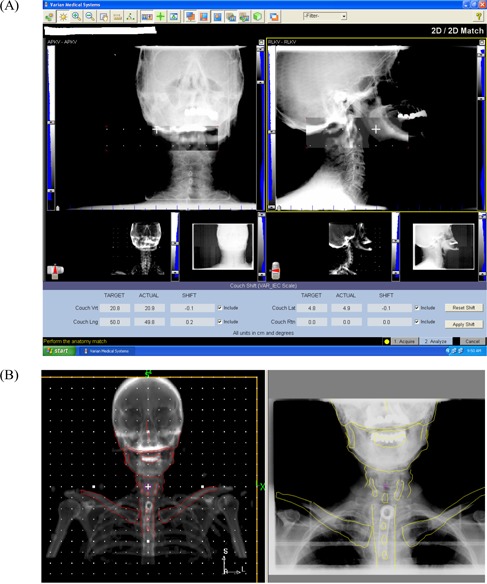
(A) The registration window of the Varian On‐Board Imager (Varian Medical Systems, Palo Alto, CA), and (B) the offline template matching window.

Translational setup error results are based on registrations done at the OBI console. Image registration on the OBI unit is performed by alignment of the bony anatomy in the overlaid reference and OBI images. Alignment can be evaluated either by image overlay (in which the reference and OBI images are overlaid in gray scale or color and aligned) or by a “spyglass” method in which a movable inner window separates the reference digital reconstructed radiograph (DRR) on the inside from the OBI image on the outside. Alignment is evaluated by examining the continuity of bony structures across the edges of the inner window. Fig. 1(A) shows the OBI console with the spyglass method in use.

Table 1 shows relevant patient information. Our study was performed while the remote couch correction feature was still being commissioned, and therefore, for 5 of the 7 patients (denoted by a superscript in Table 1), the shift suggested by the OBI was not applied. The shifts suggested by the OBI were instead analyzed offline and were used to determine a weekly setup shift analogous to the standard weekly setup shift determined by MV portal imaging (in which a single MV orthogonal pair is used to determine the setup shift for the following week). In this case, the weekly setup shift was determined by calculating the average shift of the most recent 3 – 5 OBI imaging sessions. Shifts less than 2 mm in any direction were not applied.

**Table 1:** Patient information

		Fractions	
Patient	Site	Treated	Imaged
1^a^	Larynx	33	12
2^a^	Trachea	25	13
3^a^	Nasopharynx	33	15
4	R cerebellum	5	5
5^a^	Parotid	33	13
6^a^	Thyroid	35	11
7	Base of tongue	33	10

a Patients for whom the weekly correction protocol was used.

Online correction was carried out for the remaining 2 patients. One of these 2 patients was the patient receiving hypofractionated treatment (patient 4 in Table 1). That patient had online correction for every treatment. The other patient (patient 7 in Table 1) was conventionally fractionated and received online correction on imaging days. Because of a long treatment time, this patient had difficulty tolerating the treatment. We therefore decided to discontinue this imaging protocol approximately halfway through treatment.

Patients were imaged 3 times weekly on average. On an OBI imaging day, the patient was set up to skin marks using a mask that immobilized the head and shoulders (Orfit Industries, Wijnegem, Belgium). One patient did not have shoulder immobilization. An initial shift, $s_{\text{init}}$, if the patient had one, was applied. The initial shift was recorded on the OBI data sheet. An OBI orthogonal image pair was then acquired, and bony registration was subsequently used to perform a manual registration between the OBI images and the corresponding reference images. (Auto‐registration by the OBI was not used for this study.) This registration was recorded as the OBI shift, $s_{\text{OBI}}$.

Because 5 patients were handled with a weekly setup shift and 2 patients with online correction, we report two types of interfractional setup error:

- The interfractional translational setup error without any shifts is given by $s_{\text{init}}+s_{\text{OBI}}$ for the 5 patients with an initial shift, and by $s_{\text{OBI}}$ alone for the 2 patients with online correction.
- The residual error for the 5 patients who had an initial shift is given simply by $s_{\text{OBI}}$.

The first set of results represents the setup error that would have occurred had the patient simply been set up to cast lines and treated. Those results therefore evaluate the reproducibility of the initial setup and the mask. The second set of errors represents the remaining (residual) setup error associated with the weekly correction protocol based on recent OBI images.

After the treatment, the patient was re‐imaged, and another OBI registration was performed. The difference between the post‐treatment and pretreatment OBI shifts constituted the intrafractional setup error for that day. If, after the treatment, discomfort led to the patient being unable to maintain position on the treatment table any longer, the post‐treatment scan was not performed. Therefore not all of the imaging days included post‐treatment acquisitions. The 2 patients that had online corrections may have received more than 1 OBI image pair before treatment to confirm the shifts that were applied. In cases such as these, the post‐treatment OBI image pair was compared to the *final* pretreatment image pair to determine intrafractional error. All OBI images were saved in the in‐house picture archiving and communication system for retrospective review by the physician and for offline analysis.

### B. Rotational setup error

The OBI console did not permit measurement of rotational error in both the sagittal (right lateral) and coronal (anterior–posterior) views; however, we were able to measure this error using a template matching method in the in‐house software. Reference images were opened in the in‐house registration program and anatomic landmarks were delineated with a contouring tool. Registration was performed by superimposing the landmark contours from the reference image onto the OBI image after the isocenters had been aligned. The landmark contours were rigidly translated and rotated as a whole until the best match was achieved between the contours and the corresponding anatomy on the OBI image. The rotational component of this registration constituted the rotational setup error. Fig. 1(B) shows the console with this method in use.

Data for patient 7 were not available for offline analysis, and therefore rotation was not measured for this patient. A low isocenter meant that the skull and hard palate of patient 6 were not visible on the sagittal OBI images, and therefore a meaningful measurement of head rotation in that plane was not possible. Consequently, rotation in only the coronal plane is reported for patient 6. The step size in rotational measurement for our offline software was 0.5 degrees.

Rotational setup error in the sagittal view was measured primarily using the cervical spine, hard palate, and base of skull as landmarks. Rotational error in the coronal view was evaluated primarily using the cervical spine, mandible, and (when visible) septum. The entire image was rigidly rotated, and the best match between the reference and OBI image was chosen. The measured rotational errors may therefore not be the same as non‐rigid “head rotation,” which has been measured as rotation around C2 in a recent paper by Zhang et al.^(11)^

### C. Uncertainty

To estimate the uncertainty resulting from various registration techniques, the in‐house template‐matching registration software described in the previous subsection was used offline to independently measure the translational setup error for patients 1 – 6. The resulting setup errors were compared with the corresponding measurements at the OBI console.

To estimate inter‐observer variability, another physicist independently determined translational setup error for patient 1 (22 orthogonal pairs) and rotational error for patient 3 (26 orthogonal pairs) using the in‐house software. Pretreatment and post‐treatment OBI images were both used in the comparison.

### D. Statistics

For each patient, setup error data were tested to determine if the mean setup error in each direction was significantly different from zero. Because the sample sizes were small, it was assumed that the distribution of $\left(M-\mu )/\text{sqrt}(s^{2}/n\right)$, where *M* is the sample mean, μ is the mean of the parent distribution, $s^{2}$ is the sample variance, and *n* is the number of data points in the sample, is given by a *t* distribution with *n* – 1 degrees of freedom. Given that assumption, we tested the hypothesis that μ=0 for each sample.^(12)^

## III. RESULTS

### A. Translational setup error

**Figure 2:**
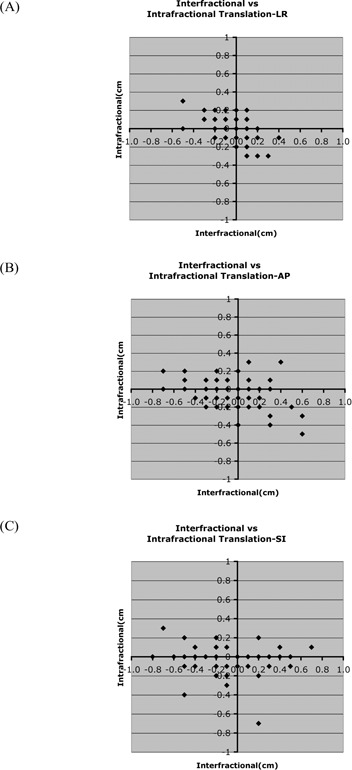
Intra‐ versus interfractional translational error for the patient group in the (A) right–left (RL), (B) anterior–posterior (AP), and (C) superior–inferior (SI) directions.

Table 2 lists the interfractional translational error, and Fig. 2 plots it against intrafractional error. A positive number indicates a setup error in the left, anterior, or superior direction. Of the 7 patients, 6 had nonzero systematic errors in the superior–inferior (SI) direction (p<0.05), and 5 had nonzero systematic errors in the anterior–posterior (AP) direction (p<0.05). Of the 6 patients with nonzero SI errors, 5 errors occurred in the inferior direction. No patients had nonzero systematic errors in the right–left (RL) direction. The average (±standard deviation) interfractional translational error of the pooled patient data was 0.0±0.2 cm, −0.1±0.3 cm, and −0.2±0.3 cm in the RL, AP, and SI directions respectively.

**Table 2:** Interfractional translational setup error, average ± standard deviation (minimum/maximum)

Patient	RL	AP	SI	Data points
1	−0.1±0.1	−0.3±0.2 ^a^	0.4±0.2 ^a^	10
	(−0.3/0.2)	(−0.5/0.1)	(0.2/0.7)	
2	−0.1±0.2	−0.3±0.1 ^a^	0.0±0.2	13
	(−0.5/0.1)	(−0.5/−0.1)	(−0.3/0.2)	
3	0.0±0.2	0.3±0.3 ^a^	−0.2±0.2 ^a^	13
	(−0.3/0.4)	(−0.2/0.6)	(−0.7/0.0)	
4	0.1±0.1	0.2±0.1 ^a^	−0.4±0.1 ^a^	6
	(0.0/0.3)	(0.1/0.3)	(−0.6/−0.3)	
5	0.0±0.1	−0.1±0.1 ^a^	−0.1±0.1 ^a^	12
	(−0.3/0.2)	(−0.3/0.1)	(−0.3/0.2)	
6	−0.1±0.2	0.0±0.3	−0.4±0.2 ^a^	11
	(−0.4/0.1)	(−0.7/0.3)	(−0.8/0.2)	
7	0.0±0.3	−0.3±0.4	−0.5±0.4 ^a^	10
	(−0.5/0.4)	(−0.7/0.4)	(−0.9/0.3)	
Pooled	0.0±0.2	−0.1±0.3	−0.2±0.3	72
	(−0.5/0.4)	(−0.7/0.6)	(−0.9/0.7)	
∑	0.1	0.2	0.3	
$\sigma_{\text{RMS}}$	0.2	0.2	0.2	

a Presence of systematic errors significantly different from zero (p<0.05).

∑ = the population standard deviation of the systematic shifts; $\sigma_{\text{RMS}}$ = the root mean square of the random errors, calculated for comparison purposes.

For comparison purposes, the population standard deviation of the individual patient systematic error ∑ and the root mean square of the individual patient random errors $\sigma_{\text{RMS}}$ was calculated for comparison with recent work by other institutions. Table 2 also gives these results.

Table 3 shows the residual error associated with the weekly setup correction protocol for the 5 patients for which it was used. This residual error arises in part from systematic error (in which the average setup error over the week is not equal to the setup shift being used for that week) and random error (day‐to‐day fluctuations around that average). After application of the setup correction protocol used for these 5 patients, SI errors significantly different from zero were found for only 3 patients, and AP errors significantly different from zero were found for just 1 patient.

**Table 3:** Residual interfractional setup error for 5 patients who used a weekly setup shift, average ± standard deviation (minimum/maximum)

Patient	RL	AP	SI	Data points
1	−0.1±0.1	−0.2±0.2 ^a^	0.2±0.2 ^a^	10
	(−0.3/0.2)	(−0.5/0.1)	(−0.1/0.5)	
2	−0.1±0.2	−0.1±0.2	0.0±0.2	13
	(−0.5/0.1)	(−0.5/0.2)	(−0.3/0.2)	
3	0.0±0.2	0.0±0.4	−0.2±0.2 ^a^	13
	(−0.3/0.4)	(−0.5/0.6)	(−0.6/0.2)	
5	0.0±0.1	−0.1±0.2	−0.1±0.1 ^a^	12
	(−0.3/0.2)	(−0.3/0.3)	(−0.3/0.2)	
6	−0.1±0.2	0.1±03	0.1±0.3	11
	(−0.4/0.1)	(−0.2/0.7)	(−0.4/0.7)	
Pooled	−0.1±0.2	0.0±0.3	0.0±0.2	59
	(−0.5/0.4)	(−0.5/0.7)	(−0.6/0.7)	
∑	0.0	0.1	0.1	
$\sigma_{\text{RMS}}$	0.3	0.3	0.2	

a Presence of systematic errors significantly different from zero (p<0.05).

∑ = the population standard deviation of the systematic shifts; $\sigma_{\text{RMS}}$ = the root mean square of the random errors, calculated for comparison purposes.

Table 4 lists the intrafractional translational setup error, and Fig. 2 plots it. As can be seen from the figure, intrafractional translational setup error was smaller than its interfractional counterpart. No patients had systematic intrafractional translational errors significantly different from zero.

**Table 4:** Intrafractional translational setup error for each patient^a^ and for pooled data, average ± standard deviation (minimum/maximum)

Patient	RL	AP	SI	Data points
1	0.0±0.1	−0.1±0.1	0.0±0.1	10
	(−0.3/0.2)	(−0.2/0.2)	(−0.1/0.2)	
2	0.0±0.1	0.0±0.1	0.0±0.1	12
	(−0.2/0.3)	(−0.2/0.1)	(−0.1/0.2)	
3	0.0±0.1	−0.1±0.3	−0.1±0.2	10
	(−0.3/0.2)	(−0.5/0.3)	(−0.3/0.3)	
4	0.0±0.2	0.0±0.1	0.0±0.0	5
	(−0.3/0.2)	(−0.2/0.1)	(−0.1/0.0)	
5	0.0±0.1	0.0±0.1	0.0±0.1	10
	(−0.2/0.2)	(−0.2/0.2)	(−0.2/0.1)	
6	0.0±0.1	−0.1±0.1	−0.1±0.3	9
	(0.0/0.2)	(−0.3/0.2)	(−0.7/0.2)	
7	−0.1±0.1	0.0±0.1	0.0±0.0	6
	(−0.2/0.0)	(−0.2/0.1)	(0.0/0.0)	
Pooled	0.0±0.1	−0.1±0.2	0.0±0.1	69
	(−0.3/0.3)	(−0.5/0.3)	(−0.7/0.3)	

a No patients had systematic errors that were significantly different from zero.

### B. Rotational setup error

**Figure 3:**
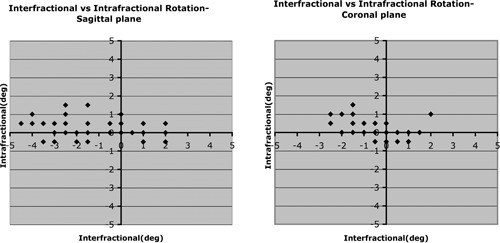
Inter‐ and intrafractional rotation for patients 1 – 5 (sagittal view) and 1 – 6 (coronal view). Rotation was not measured for patient 7. “Sagittal view” refers to a right lateral image; “coronal view” refers to an anterior–posterior image.

Table 5 lists the interfractional rotational setup error, and Fig. 3 plots it. For each view, a positive error indicates clockwise rotation. Of the 5 patients measured for sagittal rotation, 4 showed systematic errors significantly different from zero (p<0.05). Of the 6 patients measured for coronal rotation, 3 had systematic errors significantly different from zero (p<0.05). The rotational error for the pooled data was −1.1±1.7 degrees in the sagittal view and −0.5±0.9 degrees in the coronal view.

**Table 5:** Interfractional rotational setup error for patients 1 – 5 and for pooled data, average ± standard deviation (minimum/maximum)

Patient	Sag	Cor	Data points
1	−1.5±0.7 ^a^	−0.2±0.7	12
	(−2.5/0.0)	(−1.5/0.0)	
2	−0.2±0.5	0.1±0.8	13
	(−1.5/0.5)	(−1.0/2.0)	
3	−2.9±0.9 ^a^	−1.3±0.8 ^a^	14
	(−4.5/−1.5)	(−2.5/0.0)	
4	−2.8±0.9 ^a^	0.3±0.4	6
	(−4.0/−1.5)	(−1.5/0.0)	
5	1.3±0.8 ^a^	−0.8±0.4 ^a^	12
	(0.0/2.0)	(−1.5/0.0)	
6	___^a^	−0.6±0.7 ^a^ (−1.5/0.0)	10
Pooled	−1.1±1.7	−0.5±0.9	57
	(−4.5/2.0)	(−2.5/2.0)	

a Presence of systematic errors significantly different from zero (p<0.05).

b Images from the Varian On‐Board Imager (Varian Medical Systems, Palo Alto, CA) did not include enough of the head to measure rotation with reasonable accuracy. Rotation was not measured for patient 7.

Intrafractional rotation was also small relative to interfractional rotation (Table 6, Fig. 3). The systematic intrafractional rotation of the pooled data was measured as 0.3±0.6 degrees and 0.2±0.5 degrees in the sagittal and coronal views respectively. No patient had intrafractional systematic errors significantly different from zero.

**Table 6:** Intrafractional rotational setup error for each patient and for pooled data, average ± standard deviation (minimum/maximum)

Patient	Sag	Cor	Data points
1	0.4±0.8	0.2±0.6	10
	(−0.5/1.5)	(−0.5/1.5)	
2	0.1±0.2	0.2±0.4	12
	(0.0/0.5)	(−0.5/1.0)	
3	0.3±0.6	0.3±0.6	11
	(−0.5/1.5)	(−0.5/1.0)	
4	0.2±0.6	−0.2±0.3	5
	(−0.5/1.0)	(−0.5/0.0)	
5	0.3±0.5	0.2±0.4	10
	(−0.5/1.0)	(−0.5/0.5)	
6	___^b^	0.0±0.3	9
		(−0.5/0.5)	
Pooled	0.3±0.6	0.2±0.5	48
	(−0.5/1.5)	(−0.5/1.5)	

a No patients had systematic errors that were significantly different from zero.

b Images from the Varian On‐Board Imager (Varian Medical Systems, Palo Alto, CA) did not include enough of the head to measure rotation with reasonable accuracy. Rotation was not measured for patient 7.

### C. Uncertainty because of registration technique

**Figure 4:**
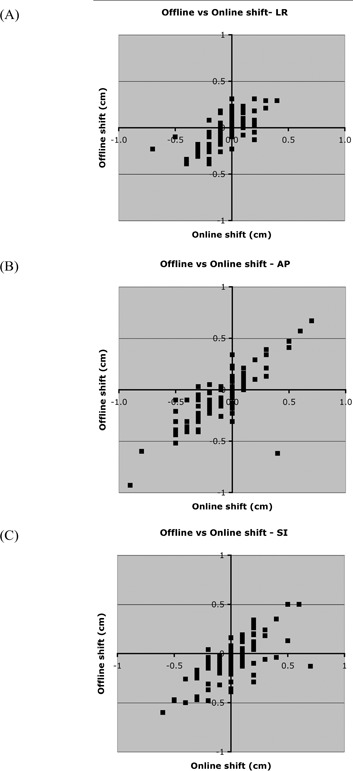
Comparison of registration techniques for (A) right–left (RL), (B) anterior–posterior (AP), and (C) superior–inferior (SI) directions. “Offline shift” refers to the in‐house template‐matching software; “online shift” refers to the Varian On‐Board Imager (Varian Medical Systems, Palo Alto, CA). The SI shift for the offline software is the average of the shifts measured in the coronal and sagittal views.

Translational shifts for 5 of the 7 patients studied were independently measured from the OBI images using the in‐house template matching software. Fig. 4 and Table 7 show the results. The average difference was within 0.1 cm in any direction, although outliers as large as 1.0 cm were seen. The SI shift for the offline software was the average between the SI shift measured in the sagittal view and the SI shift measured in the coronal view. The SI shifts in the OBI software (online) were linked—that is, changing the SI registration in one view also changes it in the other.

**Table 7:** Results of registration technique and inter‐observer comparisons^a^ for translation and rotation

Quantity	Registration technique	Inter‐observer difference
RL translation (cm)	0.0±0.1	0.0±0.1
	(−0.3/0.5)	(−0.1/0.4)
AP translation (cm)	0.0±0.2	0.0±0.1
	(−1.0/0.4)	(−0.1/0.1)
SI translation (cm)	−0.1±0.2	Sag:0.1±0.1
	(−0.8/0.2)	(0.0/0.4)
		Cor:−0.2±0.1
		(−0.4/0.1)
Rotation, sagittal view (deg)	—	0.3±0.9
		(−1.0/2.5)
Rotation, coronal view (deg)	—	1.1±1.4
		(−0.5/5.0)

a Results from the Varian On‐Board Imager (OBI: Varian Medical Systems, Palo Alto, CA) were compared with the offline software results for the same user. Rotations were excluded from this comparison because they were not measurable with the OBI. The inter‐observer comparison was performed on the offline software only because the OBI could not be used retrospectively.

### D. Inter‐observer variability

**Figure 5:**
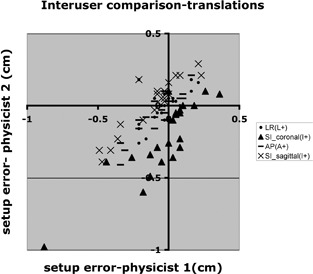
Inter‐observer comparison for right–left (RL), anterior–posterior (AP), and superior–inferior (SI) directions. The SI comparison is divided into the SI measurements from the sagittal view and the coronal view.

For patient 1, 2 physicists used the in‐house analysis software to independently measure translational errors. Fig. 5 and Table 7 show the results.

Each image from the orthogonal pair is registered independently using the offline software as opposed to the OBI software (in which the two images are registered simultaneously). Therefore, for the offline software, an SI shift is reported for both the AP (coronal) view and the RL (sagittal) view. Because of a different choice of landmark on which to register, a small systematic difference was seen between the 2 physicists with regard to the SI shift for the AP view (Fig. 5).

**Figure 6:**
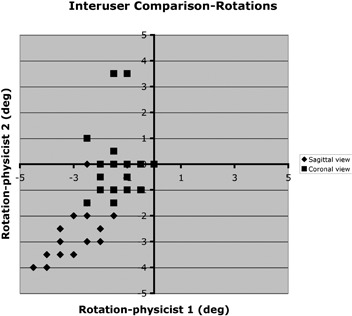
Inter‐observer comparison for rotation. Pre‐ and post‐treatment images are both included. “Sagittal view” refers to a right lateral image; “coronal view” refers to an anterior–posterior image.

The offline software was also used to compare measurements of rotational setup error for patient 3 between the 2 physicists (Fig. 6, Table 7). Small systematic differences were seen in the coronal view, again because of a different choice of landmarks by the 2 users.

### E. Total uncertainty

Based on these data, the inter‐observer uncertainty in translational setup error, expressed as the standard deviation of the difference between the 2 users, is 0.1 cm in each direction. After adding the uncertainty associated with the registration technique, the total translational registration uncertainty in the present study is estimated to be 0.2 cm in each direction. Based on the inter‐observer study of rotational error, the estimated uncertainty is 1.0 degree in the sagittal view and 1.5 degrees in the coronal view.

### F. Correlation between translational and rotational errors

A very weak correlation $\left(R^{2}=0.38\right)$ was seen between negative AP isocenter shifts and positive rotation in the sagittal view, indicating that, when the neck is not sufficiently extended, a posterior shift tends to be applied.

## IV. DISCUSSION

Because of the increasing need for precision in dose delivery, higher prescription doses, and the introduction of larger treatment volumes, head‐and‐neck setup has become more critical. With the advent of image‐guided radiotherapy (IGRT) and its associated imaging hardware, head‐and‐neck setup error can be measured with lower doses than would be required with comparable MV imaging.

The present study was performed mainly while the remote couch correction function of the OBI was being tested, and therefore, for 5 patients, a weekly setup shift was used based on the most recent 3 – 5 OBI imaging sessions. Given the circumstances, our study is not meant to illustrate the routine clinical use of OBI, which is to correct setup discrepancies online. Rather, we intended to use the imaging and registration capabilities of the OBI unit to study setup error.

Because the same setup protocol was not used for all 7 patients, the interfractional translational error without shift was reported, effectively giving the setup error associated with simply setting up the patient to cast lines and treating. These results also represent the shift that would have been applied had daily online OBI correction been used without any initial setup shifts. Most of the patients had small nonzero systematic errors in the inferior direction, which could be associated with insufficient neck extension (although that supposition is not certain). Head‐and‐neck patients at our institution are set up using biangulation tattoos; head rotation is set by the mask. Lateral images are more reliable when assessing SI alignment, but if the AP image is used as well, a slight head rotation forward could be interpreted as an inferior setup error.

Because 5 of the 7 patients received a weekly setup shift based on recent OBI sessions, we here report the residual error associated with that setup protocol as well. Such a protocol would be expected to single out systematic errors more effectively than does our standard setup protocol for non‐image‐guided head‐and‐neck patients: namely, a weekly setup shift determined from a single imaging session at the beginning of the week.

Random error is still present and cannot be significantly reduced except by daily imaging. In the case of daily imaging, it would be expected that the residual error would be a function of the action level chosen for shifting the patient and of intrafractional motion after the shift is applied. Here, we used an action level of 2 mm for the weekly shifts. Patients 1 and 6 had average SI errors of 0.4 cm. These errors were reduced to 0.2 cm after correction. Furthermore, for these 5 patients, no average setup error exceeded 0.2 cm after correction.

In our study, interfractional setup error was larger than intrafractional setup error both in terms of translation and of rotation. That finding suggests that the mask alone is more effective at limiting patient motion during the treatment than it is in reproducing the setup. Daily pre‐treatment imaging and correction, which is the standard clinical use for OBI, could reduce residual interfractional setup error and make it comparable with intrafractional error.

Other studies evaluating head‐and‐neck setup error have shown comparable results with respect to interfractional translation (Table 8). Zhang et al.^(11)^ used a CT‐on‐Rails system to measure ∑ and $\sigma_{\text{RMS}}3$ times weekly throughout treatment at various anatomic landmarks. Another CT study by Guckenberger et al.^(9)^ used automated bony matching of cone‐beam CT to measure setup error in 8 head‐and‐neck patients. The results from that study are tighter than our results are, possibly (but not definitely) as a result of reduced measurement uncertainty. Hong et al.^(8)^ measured setup error for 10 head‐and‐neck patients with a precision, optically guided patient localization system. Using implanted gold markers, van Asselen et al.^(6)^ monitored head‐and‐neck setup error in 10 patients. Linthout et al.^(10)^ used three‐dimensional (3D) fusion of X‐ray images with DRRs.

**Table 8:** Comparison of results for interfractional translational setup error (SE)^a^ with other studies, alphabetically by first author, present study first

	SE (cm)			∑			σ (cm)		
Reference	RL	AP	SI	RL	(cm) AP	SI	RL	AP	SI
Mechalakos et al.	0.0±0.2	0.1±0.3	0.2±0.3	0.1	0.2	0.3	0.2	0.2	0.2
Guckenberger et al.^(9)^	0.1±0.1	0.1±0.1	0.1±0.2	—	—	—	—	—	—
Hong et al.^(8)^	0.1±0.4	0.2±0.5	0.0±0.3	—	—	—	—	—	—
Linthout et al.^(10)^	0.0±0.1	0.1±0.1	0.0±0.2	—	—	—	—	—	—
Van Asselen et al.^(6)^	—	0.1	0.0	—	0.1	0.2	—	0.1	0.1
Zhang et al. (C2)^(11)^	0.1	0.1	0.1	0.3	0.2	0.2	0.2	0.2	0.2

a Pooled data. Numbers are rounded to the nearest 0.1 cm, and only magnitudes are given for comparison purposes. RL=right–left; AP=anterior–posterior; SI=superior–inferior; ∑ = population standard deviation of the systematic shifts; σ = root mean square or average of the random errors.

Kim et al.,^(7)^ using a combination thermoplastic mask and bite block apparatus for setup, kept intrafractional translation within 0.15 cm for 95% of the treatment time of IMRT head‐and‐neck patients and central nervous system patients. Linthout et al.,^(10)^ using infrared tracking, measured intrafraction motion of approximately 0.1 cm standard deviation in each direction. Based on the standard deviations in the three directions of the pooled intrafractional motion data, we estimate the uncertainty associated with intrafractional motion to be 0.2 cm.

In our study, most patients had a nonzero systematic rotational error in the sagittal view. Possible explanations for this error include the fact that these patients are initially set up using biangulation of skin marks, and as mentioned earlier, it is left to the mask to set the correct sagittal rotation. If the rotational error suggested head rotations alone, an average of −1.1 degrees in the sagittal view would suggest that the head was overextended, which contradicts the hypothesis that the head is too far forward as suggested by the inferior translational shifts discussed earlier. However, rotations in this study were measured primarily using the cervical spine and the inferior part of the head. (The field of view of the OBI did not allow visualization of the entire head.) Whether a negative rotation necessarily represents a head rotation to the rear is therefore not clear.

Table sag plays a role, given that the head‐and‐neck board on which the patients lie extends from the edge of the treatment couch and can bend by as much as 1 – 1.5 degrees, based on measurements of some patients in the treatment room. On the other hand, the CT simulator has no sag, and therefore neither does the reference CT image.

Rotational error obviously cannot be cured by translation alone, but its effects can be minimized—for example, by selectively registering the area of interest. The treatment team must determine if the severity of rotation is large enough to warrant manipulating the patient before commencing treatment. In our case, manipulation was rare; however, it did occur in some cases—particularly that of patient 7, because of a spinal curvature seen in the coronal (AP) image. For this patient, a number of imaging sessions called for repeated manipulation, and to limit the time that the patient remained on the table, the post‐treatment images were not acquired. That situation illustrates one of the inherent features of IGRT. A decision must be made about what to do with the additional information resulting from the use of higher‐resolution imaging. Some differences may not have been as prominent had traditional MV imaging been used. Bony resolution is not as good in such images, and the imaging is less frequent.

Table 9 compares the interfractional and intrafractional rotational setup errors found in the present study and in others. Those results are also comparable. Linthout and colleagues^(10)^ reported ranges of 8.2 degrees in the sagittal view and 4.1 degrees in the coronal view, which were larger than our ranges of 2.0 degrees in both views. As mentioned earlier, our registration is more coarse (0.5‐degree steps). Beyond the apparently larger uncertainty of our method for measuring intrafractional rotation as compared with the method used by the Linthout group (3D registration of DRRs with X‐ray images), the reason for the larger range in intrafractional motion in the Linthout data is not clear. Inherent biases and increased subjectivity in human observation versus automatic registration may also contribute to the differences in measured rotation. A future work comparing these two methods would be illuminating.

**Table 9:** Comparison of results for interfractional and intrafractional rotational error^a^ with other studies, alphabetically by first author, present study listed first

	Rotation (degrees)			
	Interfractional		Intrafractional	
Reference	Sagittal	Coronal	Sagittal	Coronal
Mechalakos et al.	1.1±1.7	0.5±0.9	0.3±0.6	0.2±0.5
Guckenberger et al.^(9)^	0.7±1.5	1.1±1.7		
Hong et al.^(8)^	0.5±2.3	0.5±1.6		
Linthout et al.^(10)^			0.2±0.8	0.1±0.6
Zhang et al.^(11)^	1.0±2.0	0.2±1.0		

a Results are rounded to the nearest 0.1 degree, and only magnitudes are given for comparison purposes.

Three different methods are available for registering these images:

- grayscale overlay, in which the acquired image is overlaid with the reference image and the images are manipulated until they lie atop each other (as the OBI does for translational setup errors).
- template matching, in which contours are drawn on the reference image and these contours are overlaid with the acquired image and matched to the anatomy (as our offline software does).
- grid overlay, in which images are viewed side by side with a grid overlaid on both. (This method was not used in the present study, although it is fairly standard in clinics without IGRT tools.)

In the present study, we saw no systematic difference between the first two methods; however, differences occurred from case to case. Those differences are attributable in part to preferential registration of various parts of the image in the case of deformation such as a head rotation. Also, the inter‐observer study showed that the region of interest can have an effect on registration.

Overall, based on comparisons of registration technique and inter‐observer comparisons, we estimate the uncertainty in registration to be approximately 0.2 cm and 1.0 – 1.5 degrees. Pisani et al.^(5)^ reported that average inter‐observer variability, expressed as the average difference between users when template alignment is being used to register the same set of data, was less than 0.2 cm in all three directions for both the head and the neck area. The average difference for our data was also no larger than 0.2 cm, which is a comparable result.

Translational setup error measurements in our study were made by aligning either DRRs (in the case of the OBI software) or contours (in the case of the in‐house software) with OBI images. One limitation of such a technique is that the user must, in some cases, find a “best fit” if deformation in the patient position has occurred. The same limitation applies to rotation measurements using the offline software. Besides the inherent subjectivity of such a measurement, no information is conveyed about the degree of deformation, if any, that is present. It should therefore not be implied that, if a particular setup error ε is reported, the deviation in other parts of the image is limited to ε. Occasionally, images were obtained for which the setup error may have been acceptable given a visual “best fit,” but deviations in parts of the image were larger. Zhang et al.^(11)^ found variability in setup error when different regions of interest were isolated. A separate study of deformation for this data set is planned.

## DISCLOSURE

The work reported here was presented in part at the 47th Annual Meeting of the American Society for Therapeutic Radiology and Oncology in Denver, Colorado, October 2005.
